# Percutaneous versus open release for trigger finger: a retrospective comparison of outcomes, complications, and costs in 110 patients from a Swedish cohort

**DOI:** 10.1186/s13018-026-07005-w

**Published:** 2026-06-09

**Authors:** Cecilia Stalberg Ostwald, Anders Henricson, Daniel Muder

**Affiliations:** 1 Department of Surgical Sciences/Orthopaedics and Hand Surgery, Uppsala University, Entrance 70, 75185 Uppsala, Sweden; 2 Centre for Clinical Research Dalarna - Uppsala University, Nissers väg 3, 79182 Falun, Sweden

**Keywords:** Trigger finger, Tenosynovitis, Tenosynovectomy, Minimally invasive surgical procedure, Postoperative complications

## Abstract

**Background:**

Trigger finger (TF) is a common hand condition often requiring surgery. Open surgery (OS) is the standard treatment, with high success rates. Percutaneous release (PR) offers potential advantages regarding cost, procedure time, and recovery, although its effectiveness remains debated in different clinical settings. This study compares outcomes, complications, and resource use between PR and OS in a Swedish hospital setting where PR was newly introduced.

**Methods:**

We conducted a retrospective observational study including 110 patients treated for TF between 2018 and 2020 at the Department of Orthopaedics, Falu Regional Hospital, Falun, Sweden. Fifty fingers underwent PR and 94 fingers were treated with OS. PR was performed by two surgeons with procedure-specific expertise corresponding to level 1 and 2, respectively. The primary outcome was elimination of triggering, evaluated through follow-up visits or phone interviews at a median of 39.5 months (interquartile range 13) post-treatment. Secondary outcomes included complications, additional treatments, DASH scores, extension deficits, and estimated treatment costs.

**Results:**

Triggering was resolved in 26/50 (52%) fingers treated with PR and 87/94 (93%) treated with OS (*p* < 0.001). Secondary interventions were required in 19 (38%) PR cases versus 2 (2%) in the OS group. No major complications (e.g. neurovascular or tendon injury) were reported. Minor transient symptoms (e.g. tenderness, scar pain) were reported by 8% of PR patients and 6% of OS patients. Estimated treatment cost per patient was 53 USD for PR and 138 USD for OS. Despite lower effectiveness, 69% of PR patients indicated willingness to undergo the same procedure again, compared to 97% in the OS group.

**Conclusions:**

While percutaneous release of the A1 pulley is safe, it was significantly less effective in eliminating triggering compared to open surgery. The procedure might be more technically demanding than previously assumed. Further prospective studies are needed to clarify its cost-effectiveness in different clinical settings, e.g. when performed by less experienced surgeons.

**Level of evidence:**

Level III.

**Clinical trial registration:**

Ethical Review Board of Region Dalarna, Dnr 2020–07205, dated February 17, 2021.

**Supplementary Information:**

The online version contains supplementary material available at 10.1186/s13018-026-07005-w.

## Introduction

There is a 2.6% lifetime risk of developing a trigger finger (TF) [1], making it one of the most common indications for hand surgery [2]. TF is more prevalent in women and in patients with diabetes, and has also been associated with gout, renal disease, and rheumatoid arthritis [1, 3].

Two principal surgical treatment options exist: open surgery (OS) and percutaneous release (PR). OS is generally considered the standard treatment when non-operative measures are ineffective. The reported success rate for OS is approximately 97% [4], and in Sweden sick leave following this procedure is typically around two weeks [5].

PR was first introduced in 1958 and was considered a feasible alternative for OS, offering advantages such as avoidance of scarring, shorter recovery time, and the possibility of performing the procedure in an outpatient setting rather than in an operating room [6]. The technique involves the use of a small cutting instrument (such as a needle or needle knife) inserted through a small skin incision, without direct visualisation of the pulley [7].

Interest in minimally invasive TF release has increased during the past few decades. Variations of PR, including procedures performed with or without corticosteroid injections [8], with or without ultrasound guidance [7, 9], and using different cutting instruments, have been investigated [4, 7, 10, 11]. PR has demonstrated success rates comparable to OS, ranging from 74 to 100%, along with high levels of patient satisfaction [3, 7, 12–15]. In addition, it has been described as a resource-efficient procedure and associated with shorter recovery time and reduced duration of sick leave [1, 16].

To the best of our knowledge, there are no Scandinavian reports evaluating the outcomes of PR for TF, and OS remains the preferred treatment in most Swedish hospitals. Therefore, we aimed to assess the effectiveness and clinical applicability of PR in a Swedish healthcare setting where the technique was newly introduced.

We hypothesized that PR would be comparable to OS in terms of clinical outcomes and complications, while also offering advantages in terms of time and resource utilisation.

## Methods

### Study design and cohort

**Fig. 1:**
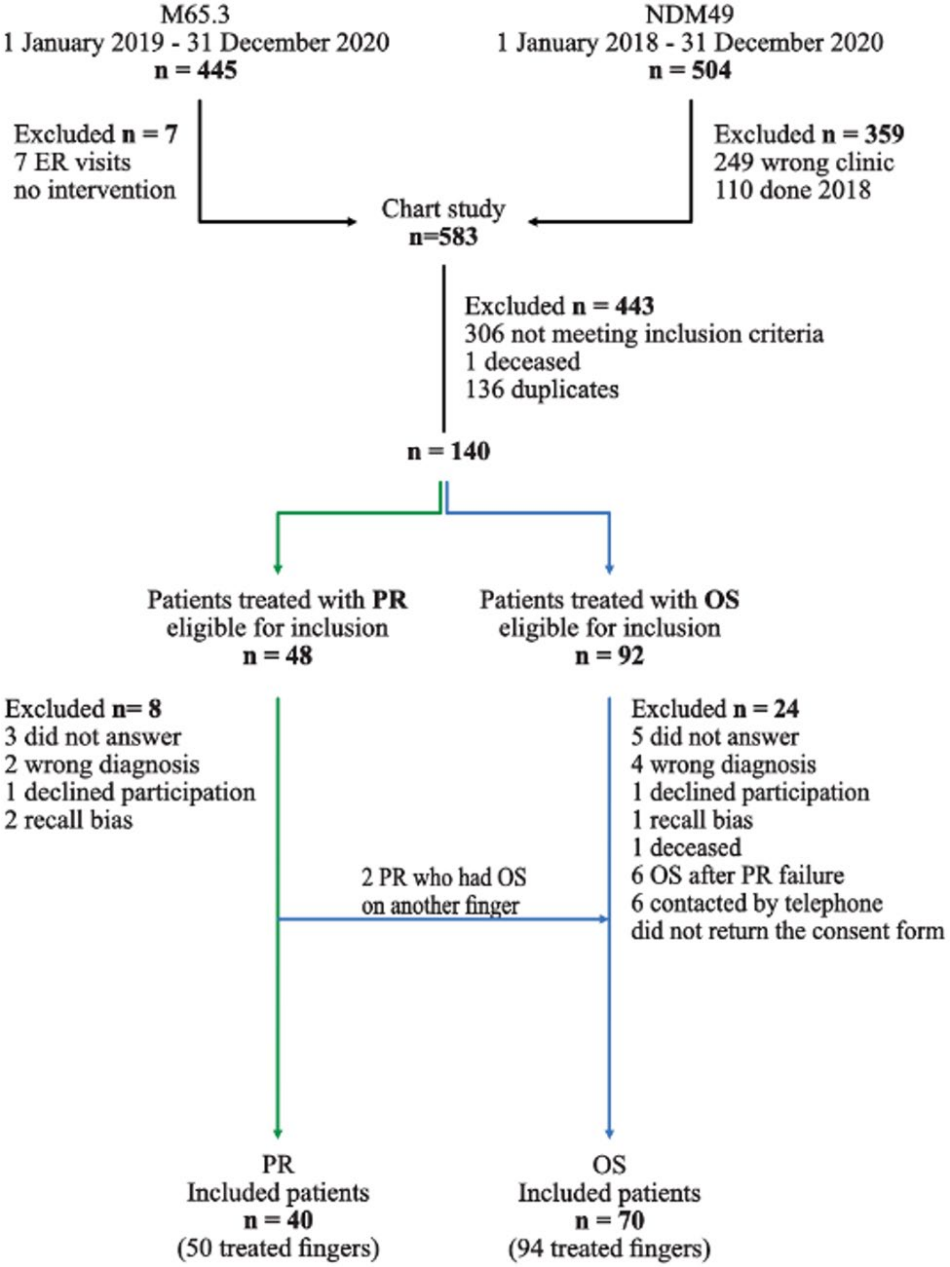
Flowchart of patient inclusion. ICD, International Statistical Classification of Diseases and Related Health Problems; ICD-code M65.3, Trigger finger; NDM49, division of tendon sheath in wrist or hand; ER, emergency room; PR, percutaneous release; OS, open surgery; n, numbers

We conducted a retrospective observational study at Falu Regional Hospital, Sweden, in 2023. The hospital’s database was searched for the International Statistical Classification of Diseases and Related Health Problems (ICD) code M65.3 (trigger finger) for the period 2019–2020, and the treatment code NDM49 (division of tendon sheath in wrist or hand) for the period 2018–2020. All patients who underwent PR for TF during the initial implementation of the technique at our clinic in 2019 were identified. Patients referred to the Department of Orthopaedics in Falun were informed about the PR procedure. PR was offered to patients willing to undergo the procedure, while those who preferred the established OS technique were scheduled accordingly. Thus, group allocation was based on patient preference rather than randomisation. To increase precision despite the limited number of patients treated with PR, we aimed to include patients in the control group (OS) in a 2:1 ratio (Fig. 1).

This study was approved by the Swedish Ethical Review Authority (Dnr. 2020–07205, dated 17 February 2021). All patients provided written informed consent. This study was completed in accordance with the Declaration of Helsinki.

### Patient recruitment

Eligible patients were provided with study information by post and scheduled for a follow-up appointment in an outpatient setting. Inclusion criteria are presented in Table 1.

**Table 1:** Inclusion criteria

Treatment for trigger finger with percutaneous release or open surgery on one or more fingers between 1 January 2019 and 31 December 2020 at our department	
Age ≥ 18 years	
No previous surgical treatment on a treated finger	
Patients could have had one or more cortisone injections prior to PR or OS	

PR, percutaneous release, OS, open surgery

Patients who declined a clinical visit were offered follow-up by telephone. Patients who did not attend their appointment and did not make contact were actively followed up by telephone. Patients were considered lost to follow-up if they did not respond after two attempts.

### Outcomes

At the follow-up, patients were asked about their perceptions of the procedure, treatment outcomes, and any subsequent interventions (see Supplementary Appendix A for the full questionnaire). Patients were asked to recall the severity of triggering prior to treatment. Based on patient recall or medical records, each treated finger was graded according to the Quinnell classification [17]. Grade 0 indicates normal tendon movement during finger flexion and extension; grade I, uneven movement; grade II, actively correctable locking; grade III, passively correctable locking; and grade IV fixed finger deformity.

The primary outcome was elimination of triggering as reported by patients after the procedure.

Secondary outcomes included Disabilities of the Arm, Shoulder and Hand (DASH) score [18] and the presence of an extension deficit of *≥* 10 degrees in either the metacarpophalangeal (MCP), proximal interphalangeal (PIP) or distal interphalangeal (DIP) joint among patients attending clinical follow-up. Additional secondary outcomes included complications (e.g. infections, neurovascular or tendon injury, and long-term pain), need for complementary treatment, and estimated treatment costs.

### Surgical technique

#### Percutaneous release

All PR procedures were performed by one of two surgeons: an orthopaedic specialist with expertise in hand surgery (level 3) and one orthopaedic resident (level 1), representing the range of surgical expertise at the clinic [19]. Their procedure-specific expertise with PR corresponded to level 2 and level 1, respectively.

Following hand washing and disinfection with 70% alcohol-based solution, local anaesthesia was administered to the volar aspect of the A1 pulley of the affected finger.

After sterile gloves were applied, the surgeon disinfected the skin over the A1 pulley using chlorhexidine and sterile gauze. An 18-gauge needle was then inserted percutaneously over the pulley, and the A1 pulley was released using controlled sweeping movements of the needle.

Adequacy of release was assessed by repeated active flexion of the finger.

A sterile adhesive dressing was applied, and patients were advised to resume normal hand use the following day without restrictions. If required, patients were advised to use over-the-counter analgesics such as paracetamol or ibuprofen.

#### Open surgery

OS was performed under local anaesthesia using a standardised technique, involving a 1–2 cm incision at the palmar MCP crease over the A1 pulley. The wound was closed with non-resorbable sutures and covered with a sterile adhesive dressing.

As part of routine postoperative care, patients were advised to avoid lifting heavy objects (> 1 kg) for 2–3 weeks. Patients with physically demanding occupations were advised to refrain from work until suture removal and complete wound healing, typically after 2–3 weeks.

### Statistical analysis

Continuous variables are presented as means and standard deviations (SDs) for normally distributed data, and as median and interquartile ranges (IQRs) for non-normally distributed data, as assessed using Q–Q plots. Categorical variables are presented as counts and percentages. Between-group comparisons for continuous variables were performed using the independent samples t-test or the Mann-Whitney U test, based on distribution assumptions. For categorical variables, the Chi-squared test or the Mann-Whitney U test was used as appropriate, depending on whether data were nominal or ordinal. Fisher’s exact test was used when expected cell counts were < 5. A *p*-value of < 0.05 was considered statistically significant. All analyses were performed using Jamovi, (The Jamovi Project 2024).

## Results

A total of 40 patients (50 fingers) were treated with PR while the control group comprised 70 patients (94 fingers) treated with OS (Fig. 1).

Baseline characteristics were generally comparable between the two groups although the proportion of women was lower in the PR group (18/40, 45%) than in the OS group (47/70, 67%). In addition, a greater proportion of procedures in the PR group were performed by a specialist in orthopaedics (*p* = 0.015), and more treated fingers were from patients with diabetes and rheumatoid arthritis (*p* = 0.084 and 0.21 respectively) (Table 2). The median follow-up duration was 39.5 months (IQR 13) for the whole cohort.

In the PR group, 32/40 (80%) patients were treated for a single finger, 7/40 (18%) for two fingers, none for three fingers, and 1/40 (2%) for four fingers. The corresponding distribution in the OS group was 53/70 (76%), 13/70 (19%), 1/70 (1%) and 3/70 (4%), respectively.

**Table 2:** Baseline characteristics

	Percutaneous release	Open surgery	*P*-value
Number of fingers, Female sex (n, %)	50 (20, 40%)	94 (65, 69%)	< 0.001^3^*
Age, years (SD)	66.6 (10.7)	63.1 (11.7)	0.08^1^
Length of follow-up, months (IQR)	38.5 (16)	40.0 (13.5)	0.176^2^
Treatment of the dominant hand	32 (64%)	54 (57%)	0.445^3^
*Treated finger* (%)			0.195^3^
First	5 (10)	16 (17)	
Second	5 (10)	6 (6)	
Third	19 (38)	48 (51)	
Fourth	18 (36)	19 (20)	
Fifth	3 (6)	5 (5)	
*The surgeon’s level of expertise* (%)			0.015^3^*
Specialist	38 (76)	52 (55)	
Resident	12 (24)	42 (45)	
Active smoker at time of treatment (%)	8 (16)	2 missing 8 (16)	0.189^3^
*Comorbidities* (%)			
Diabetes	16 (32)	18 (19)	0.084^3^
Gout	5 (10)	7 (7)	0.598^3^
Rheumatism	11 (22)	13 (14)	0.21^3^
Kidney disease	0	3 (3)	0.552^4^
*Cortisone prior to treatment* (%)		2 missing	0.281^3^
No	16 (32)	43 (46)	
Yes, 1	19 (38)	26 (28)	
Yes, 2	9 (18)	9 (10)	
Yes, ≥ 3	2 (4)	7 (7)	
Unable to recall	4 (8)	7 (7)	
*Quinnell classification* (%)			0.679^2^
Grade 0	1 (2)	0 (0)	
Grade 1	2 (4)	13 (13)	
Grade 2	22 (44)	23 (26)	
Grade 3	22 (44)	54 (57)	
Grade 4	2 (4)	2 (2)	
Do not remember	1 (2)	0 (0)	
*Occupation type* (%)			0.141^3^
Employed	22 (44)	57 (61)	
Unemployed	0	1 (1)	
Retired	28 (56)	36 (38)	
*Type of follow-up* (%)			0.454^3^
Clinic	34 (68)	58 (62)	
Telephone	16 (32)	36 (38)	

All counts are based on number of treated fingers. Age and length of follow-up are presented as mean and standard deviation (SD) and median with interquartile range (IQR) respectively based on distribution assessed on Q-Q plots. Continuous variables were analysed using independent samples t-test (1) or Mann–Whitney U (2) test as appropriate. Categorical variables were analysed using Chi-square test (3) or Fisher’s exact test (4) when expected cell counts were <5. Quinnell classification was analysed using the Mann–Whitney U test due to its ordinal nature. The criteria for identifying patients with diabetes, gout, rheumatism or kidney disease involved receiving treatment and a formal diagnosis, * = p<0.05

Triggering was eliminated in 52% of fingers treated with PR and 93% of those treated with OS (*p* < 0.001), with an OR of 0.09 (95% CI 0.03–0.23). The proportion of patients willing to undergo the same treatment again was 69% (27/39, one missing) in the PR group and 97% (67/69, one missing) in the OS group. Additional outcomes are presented in Table 3.

**Table 3:** Outcomes

	Percutaneous release n = 50	Open surgery n = 94	*P*-value
*Primary outcome*			
Trigger elimination after intervention (%)	26 (52)	87 (93)	< 0.001^b^*
*Secondary outcomes*			
Secondary treatment following failure (n in total)	− 24	− 7	n/a
No, symptoms not severe enough	5	5	
No, but awaiting further treatment	0	0	
Yes, PR	3	0	
Yes, OS	14	1	
Yes, cortisone injection	2	0	
Yes, orthosis	0	1	
No residual symptoms at follow-up	39	75	0.80^b^
Sick leave (yes)	10 4.8 (2.9–6.7)	50 (3 missing) 14.3 (11.8–16.8)	
Sick leave duration, days (95% CI)			< 0.001^a^*
Scar pain (yes)	3	7	0.75^b^
Extension deficit (present)	12 (17 missing)	11 (40 missing)	0.10^b^
DASH score (95% CI)	15.9 (9.7–22.1) (18 missing)	13.6 (8.9–18.2) (37 missing)	0.35^a^
Overall satisfaction score (95% CI)	7.4 (6.5–8.2)	9.2 (8.9–9.5)	< 0.001^a^*
(NRS 1 worst, 10 best)			

All counts are based on treated finger. Presented as mean and 95% confidence interval (CI). Continuous non-normally distributed and ordinal data were analysed using Mann-Whitney U test (a), whereas categorical data were analysed using the Chi-square test (b). *=*p* < 0.05. Secondary treatment after failure presents how many patients with treatment failure that have had, are waiting for or don’t want secondary treatment to eliminate triggering. NRS, numerical rating scale. n, numbers.

Procedures performed by a specialist in orthopaedics accounted for 38 PR-treated fingers, with a success rate of 50%. The corresponding success rate for PR performed by a resident was 58.3%. For OS, success rates were 94.2% for specialists and 90.5% for residents.

No major complications, such as neurovascular injury or flexor tendon rupture, were observed in either group. The number of patients who recalled complications within the first postoperative week was four (8%) in the PR group and six patients (6%) in the OS group. In the PR group, transient problems included prolonged tenderness over the treatment site lasting up to three months (*n* = 2), transient pain of unspecified duration without residual symptoms at follow-up (*n* = 1), and pain lasting 2–3 weeks (*n* = 1). In the OS group, six patients reported complications in the early postoperative period. One patient was unable to recall specific details. One patient developed a granuloma requiring treatment with silver nitrate. Three patients reported unspecified wound healing issues not classified as infections, and one patient reported transient pain of unspecified duration. Another patient experienced scar pain persisting for 7–8 months. Of these six patients, five experienced transient symptoms, while one reported persistent pain at follow-up. No infections were reported in either group.

Cost estimates were based on a half-day (4-hour) treatment session. Typically, six patients were scheduled for PR during this period, compared with five patients undergoing OS. The estimated cost per patient was approximately 53 USD for PR and 138 USD for OS (Table 4), excluding any additional costs related to secondary treatment following failure.

**Table 4:** Cost estimates

Unit	Cost per unit		Percutaneous release		Open surgery	
	(USD)		*n*	Cost (USD)	*n*	Cost (USD)
Surgeon (h)	50.16		4	200.64	4	200.64
Nurse (h)	26.21		4	104.84	4	104.84
Nurse’s assistant (h)	17.77		0	0	4	71.08
Patient clothing	4.19		0	0	5	20.95
*Preoperative OS or PR procedure*						
Gloves	0.04		6	0.24	5	0.2
Sterile gloves	1.3		6	7.8	0	
Local anaesthetics	0.65		6	3.9	5	3.25
Syringe	0.05		6	0.3	5	0.25
23G needle	0.07		6	0.42	5	0.35
18G needle	0.02		12	0.24	5	0.1
Sterile cloth	0.3		1	0.3	0	
Procedure pack*	40.58		0	0	5	202.9
Surgical cap Protective mask Sterile gloves	0.55 0.05 1.28		0 0 0	0 0 0	3 10 10	1.65 0.5 12.8
Surgical blade	0.69		0	0	5	3.45
Sutures	0.72		0	0	5	3.6
Suture removal Nurse (0,5 h)	13.1		0	0	5	65.5
Total cost USD Per patient USD				318.68 53.11		692.06 138.41

Approximate costs for percutaneous release (PR) performed in an outpatient setting and open surgery (OS) performed in the operating room during a half-day session (4 h). Hourly wages for personnel were collected from Statistics Sweden search engines. Material costs were calculated based on six PR cases and five OS cases. *Procedure pack for OS includes sterile dressing, 1× suction, 2× surgical gowns, 1× sleeve protection, 10 × 12-layer gauze, 5× surgical towels nonwoven 30 × 40,15× disinfection pads nonwoven, 1× roll cotton padding 10 cm × 3 m, 1× roll elastic wrap 6 cm × 5 m, 1 × 23G needle, 1 × 18G needle, 1 × 15 blade, 1 × 10 ml syringe, 1× paper ruler 15 cm, 3× plastic bag 20 × 30 cm, 1× permanent marker. n, numbers

## Discussion

The success rate for PR reported in previous studies ranges from 74 to 100% [3, 7, 12–15]. In contrast, our findings did not confirm these results, as complete elimination of triggering was achieved in only 52% (26/50) of treated fingers.

Despite this relatively low success rate, 69% of patients in the PR group indicated that they would be willing to undergo the procedure again if they developed another TF. Patients described the procedure as straightforward and worth attempting. Conversely, patients unwilling to repeat PR primarily reported feeling unprepared for the immediacy of the procedure and discomfort associated with observing it being performed. These findings highlight the importance of thorough patient information and appropriate patient selection, particularly when introducing a new treatment modality in a setting with limited procedural experience. As reported in previous studies, patient selection is critical, the severity of triggering and symptom duration have been shown to correlate negatively to treatment success after OS [20]. However, whether these are negative prognostic factors for PR has not been established. Furthermore, socioeconomic factors have not been shown to affect the relative improvement but have been shown to affect postoperative symptom perception [21]. Consistent with previous studies, PR was associated with fewer sick leave days and no major complications (e.g., extensive scar tissue or nerve or tendon injuries) [3, 4, 11, 12].

In comparing the costs of TF release using PR or OS, we evaluated personnel and materials expenses associated with each procedure. During a half-day session, five patients can typically be treated with OS, requiring one surgeon, one nurse, and one nurse´s assistant. In contrast, six patients can be treated with PR in an outpatient setting using only one surgeon and one nurse. Based on these assumptions, the estimated cost for a half-day session was approximately 319 USD for PR and 692 USD for OS (Table 4), corresponding to a saving of approximately 85 USD per patient when PR is performed in the clinic. Marij Z. et al. reported that PR costs about 14% of OS which is lower than our estimate of 38% [1]. However, their methodology was not described in detail. The cost analysis in the present study is approximate, and future studies should include the costs of postoperative visits and additional treatments following failure, as well as formal a cost-effectiveness analyses incorporating quality-adjusted life years (QALYs).

In light of a retrospective study design, several limitations should be considered when interpreting the findings. First, outcomes were partly based on patient recall, introducing a risk of measurement bias. To mitigate this, patient responses were cross-checked against medical records when possible; however, variables such as Quinnell classification and presence of triggering were not consistently documented. PR has been recommended for Quinnell grades II-IV, as these are easier to evaluate intraoperatively rather than for grades 0-I [3]. During the procedure, patients were asked to actively flex and extend the finger to assess release; however, as triggering may vary over time, some patients who recalled having Quinnell grade II-IV symptoms may not have had reproducible triggering at the time of treatment. Second, variations in surgical technique could not be controlled for. For example, there was no documentation regarding whether MCP joint hyperextension was used, a manoeuvre that has been suggested to improve exposure of the A1 pulley volarly to increase the likelihood of complete release and reduce the risk of neurovascular injury [22]. Third, comparisons of sick leave between groups should be interpreted with caution, as postoperative recommendations differed: patients undergoing OS were routinely advised to rest for approximately two weeks, whereas PR patients were allowed immediate use of the hand. In addition, occupational demands (e.g. manual versus administrative work) may have influenced return-to-work outcomes.

Furthermore, although 110 patients were included, approximately one-third in both groups were followed up by telephone, precluding objective functional assessments and DASH scoring in these cases. This limits the interpretation of these secondary outcomes due to missing data. Patient satisfaction was assessed using a simple numerical rating scale (Likert-type), which is not a validated outcome instrument and may be influenced by factors such as the interviewer and patient expectations. While this limits the precision and comparability of satisfaction data, it provides complementary insight into patient-perceived outcomes. Another important consideration is surgeon expertise. One surgeon was a resident learning the PR technique during the study period, while the senior surgeon primarily had experience with PR for Dupuytren’s contracture, however, had limited prior experience with PR for TF. Although no formal learning curve analysis was performed due to the limited number of PR cases per surgeon, descriptive data suggest some variability in success rates between operators. Interestingly, the resident demonstrated a slightly higher success rate for PR than the specialist (58% vs. 50%), whereas the opposite pattern was observed for OS (90% vs. 94%). These findings could be due to the small sample size but may indicate that a learning curve exists for PR that is not solely dependent on overall surgical expertise [23, 24]. Moreover, we collected data on potential confounding factors such as diabetes mellitus, smoking status, previous corticosteroid injections, and other adjunctive treatments. However, these variables were not included in a formal statistical adjustment, which represents a limitation of our study.

A key strength of this study is the inclusion of a well-defined control group treated with open surgery, allowing direct comparison between PR and the current standard of care. The relatively long follow-up period, with a median exceeding three years, provides insight into the durability of each treatment effect, which is often lacking in similar studies. For example, in a meta-analysis by Zhao et al., only one included study reported follow-up beyond 30 months, with most ranging from 1 to 12 months [25].

Finally, the procedures were performed in a routine clinical setting, reflecting real-world practice and enhancing the external validity of the findings. With 110 patients included, this study represents a relatively large single-centre cohort, enabling meaningful descriptive data and analyses while acknowledging that results may differ across healthcare settings.

## Conclusion

In this study, percutaneous release of the A1 pulley was shown to be a safe procedure, but it was not as effective in eliminating triggering as OS. Although PR was performed by both a specialist and a resident, the overall success rate of 52% may suggest a potential learning curve before the success rate approaches what is typically reported for OS. Further prospective studies are needed to clarify its cost-effectiveness in different clinical settings, for example when performed by less experienced surgeons.

## Supplementary Information

Below is the link to the electronic supplementary material.

Supplementary Material 1

## Data Availability

The datasets generated and analysed during the current study are not publicly available but are available from the corresponding author on reasonable request.

## Abbreviations

TF: Trigger finger
OS: Open surgery
PR: Percutaneous release
CI: Confidence interval
ICD: International statistical classification of diseases and related health problems
MCP: Metacarpophalangeal
PIP: Proximal interphalangeal
DIP: Distal interphalangeal
DASH: Disabilities of the arm, shoulder and hand
SD: Standard deviation
QALYs: Quality-adjusted life years
