# Supplementary material for: Percutaneous versus open release for trigger finger: a retrospective comparison of outcomes, complications, and costs in 110 patients from a Swedish cohort
Source: J Orthop Surg Res. 2026 Jun 9;21:321. doi: 10.1186/s13018-026-07005-w (PMC13251119; doi:10.1186/s13018-026-07005-w)
Supplement: Supplementary file 1 — Supplementary Material 1 [file 13018_2026_7005_MOESM1_ESM.docx]

Supplementary Appendix

*A Retrospective Cohort Study Comparing Percutaneous Release with Open Surgery in 110 Patients with Trigger Finger*

**Table of contents: Page:**

Appendix A, Study-specific questionnaire…………………....................………………2-5

Appendix A

Study-specific questionnaire

Documentation support in the follow-up of patients treated for trigger finger using percutaneous needle release or open standard surgery.

**Patient's personal identification number**: __________

**Date of the completed operation according to patient medical records** *(Fill in the date and calculate the number of months)*

Fixed on: _________________. The follow-up after _____ number of months.

**Which finger was fixed and on which hand?** *(mark the correct options; if more fingers were treated, mark all of them)*

Right hand Left hand

DIG 1 - Thumb

DIG 2 - Index finger

DIG 3 - Middle Finger

DIG 4 - Ring finger

DIG 5 - Little finger

DIG 1 - Thumb

DIG 2 - Index finger

DIG 3 - Middle Finger

DIG 4 - Ring finger

DIG 5 - Little finger

**What treatment method was used?** *(identify suitable alternatives)*

Percutaneous with needle Open standard surgery

**Is it the patient's dominant hand?** *(identify suitable alternatives)*

Yes No

**Smoking?**

Yes No

**What is the patient's work?** *(Text or circle suitable alternatives)*

Works as: __________________________

Seeking work Disability pensioner, due to _________ Pensioner

**Duration of symptoms** *(circle the most suitable option)*

<6 months 6-12 months 1-2 years >2 years

**Quinell classification?** *(Circle the correct alternative. The classification refers to the movement of the tendon during flexion/extension. The underlined text is a clarification to find some correspondence in what the patient describes as discomfort)*

0 - Normal movement without hooking.

1 - Uneven movement, easier hooking, click when flexion/extension.

2 - Active correction is possible: When the finger is locked in flexion, it is still possible to extend it.

3 - Passive correction is possible: When the finger is locked in flexion, the other hand can pry it open.

4 –- Fixed deformity: cannot straighten the finger either actively or passively.

**Has the patient received treatment with a cortisone injection for the same trigger finger at any time before the above treatment?** *(Circle Yes or No and supplement the form with the number of injections of cortisone given in total to the finger(s) that the form refers to.*

Yes

Number of cortisone injections: 1 2 3 or more

No

**Has the patient received orthotic treatment on the finger prior to the procedure?** *(identify suitable alternatives)*

Yes No

**How did the anesthetic work in connection with the procedure?** *(identify suitable alternatives)*

Good Poorly Don't remember

**Were there any complications in the days/weeks after treatment?** *(e.g., bleeding, infection, aggravated pain?)*

**Has there been any triggering in treated finger(s) after the procedure?** *(identify suitable alternatives)*

Yes No

**Is the patient capable of normal hand function post-surgery? (***Circle and mark if the first answer is NO)*

Yes

No

**Problems in the first month**

**Problems in the first year**

**Still problems**

**Is the patient currently experiencing pain at the surgical site?** *(identify suitable alternatives)*

Yes

No

Every so often

**Has the patient required complementary treatment?** *(identify suitable alternatives)*

**Yes,** Percutaneous with needle Open OP Cortisone Splint

**No**

**Is the repaired finger(s) completely restored?** *(identify suitable alternatives)*

Yes

No

**Was the patient on sick leave/home from work after the procedure? (***if yes, please specify approximately how long in days)*

Yes, ________days No

**If the patient encounters problems with the trigger finger again, would they consider undergoing the same procedure?** *(Specify why the same procedure should or should not be chosen.)*

Yes, therefore

No, therefore

**Does the patient have any of the following diseases?** *(check the appropriate options)*

Diabetes Rheumatism

Gout Kidney disease

**Overall, how satisfied is the patient with the procedure and results on a scale from 1 to 10?** *(1 is very dissatisfied and 10 is very satisfied – place an X on the appropriate vertical line)*

1 5 10

**Other comments by the patient:**
